# Discovery by organism based high-throughput screening of new multi-stage compounds affecting *Schistosoma mansoni* viability, egg formation and production

**DOI:** 10.1371/journal.pntd.0005994

**Published:** 2017-10-06

**Authors:** Alessandra Guidi, Cristiana Lalli, Roberto Gimmelli, Emanuela Nizi, Matteo Andreini, Nadia Gennari, Fulvio Saccoccia, Steven Harper, Alberto Bresciani, Giovina Ruberti

**Affiliations:** 1 National Research Council, Institute of Cell Biology and Neurobiology, Campus A. Buzzati-Traverso, Monterotondo (Roma), Italy; 2 IRBM Science Park SpA Chemistry Department, Pomezia, Italy; 3 IRBM Science Park SpA, Biology Department, Pomezia, Italy; Brown University, UNITED STATES

## Abstract

Schistosomiasis, one of the most prevalent neglected parasitic diseases affecting humans and animals, is caused by the Platyhelminthes of the genus *Schistosoma*. Schistosomes are the only trematodes to have evolved sexual dimorphism and the constant pairing with a male is essential for the sexual maturation of the female. Pairing is required for the full development of the two major female organs, ovary and vitellarium that are involved in the production of different cell types such as oocytes and vitellocytes, which represent the core elements of the whole egg machinery. Sexually mature females can produce a large number of eggs each day. Due to the importance of egg production for both life cycle and pathogenesis, there is significant interest in the search for new strategies and compounds not only affecting parasite viability but also egg production. Here we use a recently developed high-throughput organism-based approach, based on ATP quantitation in the schistosomula larval stage of *Schistosoma mansoni* for the screening of a large compound library, and describe a pharmacophore-based drug selection approach and phenotypic analyses to identify novel multi-stage schistosomicidal compounds. Interestingly, worm pairs treated with seven of the eight compounds identified show a phenotype characterized by defects in eggshell assemblage within the ootype and egg formation with degenerated oocytes and vitelline cells engulfment in the uterus and/or oviduct. We describe promising new molecules that not only impair the schistosomula larval stage but also impact juvenile and adult worm viability and egg formation and production *in vitro*.

## Author summary

Schistosomiasis is a neglected disease caused by parasitic flatworms called schistosomes. The disease affects hundreds of millions of people in developing countries in the poorest tropical and subtropical regions of the world and it represents a major public health and socio-economical problem in several countries. In humans, these blood flukes reside in the mesenteric and vesicle venules. They have a life span of many years and produce hundreds of eggs daily, which are able to pass through the gut lumen or the bladder to be finally excreted into the environment for maintaining the life cycle. Part of the eggs can be trapped in host tissues inducing immunologically mediated granulomatous inflammation and fibrosis leading eventually to severe sequelae such as hepatosplenomegaly and even death. Importantly, schistosome infections increase susceptibility to other parasitic, bacterial and viral diseases. To date, essentially a single drug, praziquantel, is available to treat this parasitic disease. Despite its high tolerability and efficacy against adult parasites it has an incomplete efficacy across all stages of the *S*. *mansoni* life cycle and it does not prevent reinfection. Moreover the potential risk of drug resistance is an increasing concern. In search of novel schistosomicidal molecules we screened a large compound collection using the schistosomula, larval stage of the parasite. We identified eight novel molecules able to impair viability of schistosomula, juvenile and adult worms and also egg formation and production, two important features required for both disease transmission and progression.

## Introduction

Parasitic trematodes of the genus *Schistosoma* cause schistosomiasis, a life-threatening infectious disease affecting both humans and animals. Schistosomiasis, one of the world’s greatest neglected tropical diseases, contributes to the global morbidity with 4,026,000 DALYs (disability-adjusted life years) [1]. Among human parasitic diseases, schistosomiasis ranks second behind malaria in socio-economic terms, public health importance and prevalence in the developing world. More than 780 million people are at risk of infection and it is estimated that there are approximately 261 million infected people in 78 countries, of whom 85% reside in sub-Saharan Africa [2]. Three major species (*S*. *mansoni*, *S*. *haematobium* and *S*. *japonicum*) account for the majority of human infections. Similar to other trematodes, schistosomes have a complex life cycle consisting of both free-living and parasitic forms with several developmental stages [3]. Moreover, whereas most of the trematodes are hermaphrodites, schistosomes have evolved two separate sexes and the sexual maturation of female worms as well as the subsequent production of eggs are both dependent on the pairing status with males [4–6]. Sexually mature adult females can produce, a large number of eggs each day, formed within the ootype by one oocyte and 30–40 vitelline cells. The eggs are responsible for both parasite transmission and disease pathogenesis as part of the egg produced can be trapped into host’s tissues causing granuloma formation and inflammatory processes which interfere with organs function [7]. To date praziquantel (PZQ) is the only drug recommended for the treatment of schistosomiasis being very effective against adult worms of all the medically important *Schistosoma* species (*S*. *mansoni*, *S*. *haematobium and S*. *japonicum*) [8,9]; however, PZQ is relatively ineffective against the juvenile and schistosomula larval stages both *in vivo* and *in vitro* [10–13] and it does not prevent re-infection [14,15]. Moreover its increasingly widespread use in mass chemotherapy campaigns and the identification of field [16–19] and laboratory isolates that exhibit significantly reduced susceptibility to PZQ [20–24] represents a serious concern for the development of drug-resistance strains. Consequently the search for novel schistosomicidal compounds is currently viewed as an urgent goal and there is great interest in new chemical compounds with demonstrated (i) ability to kill the parasites, possibly targeting different developmental stages, in order to lower worms burden and (ii) capacity to impair egg production, so that pathological effects are minimized, or even completely abolished. In this work we show how we succeeded in meeting both these requirements, and identified a number of new molecules having effects on both worm viability and egg production. To meet our aims, a recently developed high-throughput assay based on ATP quantitation in the larval stage schistosomula of *S*. *mansoni* [25] was used to screen a large compound library. In the follow up step a pharmacophore-based drug selection approach and phenotypic analyses were employed to identify novel multi-stage schistosomicidal compounds. We herein describe promising new hits for further chemical optimization. These hits are active on the schistosomula larval stage as well as on juvenile and adults worms. Interestingly, treatments of worm pairs with the majority of the new hits identified, induce a phenotype characterized by defects in eggshell assembly in the ootype and egg formation with degenerate oocytes and vitelline cells engulfment in the uterus and/or oviduct. Moreover, some compounds induce also gut dilation with detachment of the gastrodermis in adult worms.

## Methods

### Reagents

Gambogic acid (GA), perhexiline maleate (1:1 racemic mixture of (R) and (S) enantiomers) (PHX), dimethylsulphoxide (DMSO), percoll, fetal bovine serum (FBS), thimerosal were purchased from Sigma-Aldrich. CellTiter-Glo (CTG) reagent, used in the schistosomula viability luminescence-based assay was from Promega. Biowhittaker Dulbecco-Modified Eagle’s Medium (DMEM) with or without phenol red, HEPES, L-glutamine were from Lonza. Antibiotic-antimycotic reagent (100x) was from ThermoFisher scientific; carmine-red and canada balsam were purchased from Merck.

### Compound library composition

At the time the high throughput screening (HTS) was performed the compound collection consisted of around 40,000 small molecules from both commercial and non-commercial suppliers. In addition to FDA- and/or EMA-approved drugs the collection contained a structurally diverse range of chemotypes with average molecular weight 370 Da. Analysis of the collection revealed an attractive distribution of physicochemical properties (e.g. logP, sp3 character and hydrogen bond donor/acceptors) and good structural diversity (average Tanimoto [26] distance from the nearest neighbour of 0.38).

### Ethics statement

Female ICR (CD-1) 4–7 week-old mice (Harlan Laboratories or EMMA) were housed under controlled conditions (22°C; 65% relative humidity; 12/12 hours light/dark cycle; standard food and water *ad libitum*). Animals were subjected to experimental protocols (Authorization N. 25/2014-PR) approved by the National Research Council, Institute of Cell Biology and Neurobiology animal care and use committee and the public Veterinary Department of the Italian Ministry of Health, and experiments were conducted according to the ethical and safety rules and guidelines for the use of animals in biomedical research provided by the relevant Italian law and European Union Directive (Italian Legislative Decree 26/2014 and 2010/63/EU) and the International Guiding Principles for Biomedical Research involving animals (Council for the International Organizations of Medical Sciences, Geneva, CH). All adequate measures were taken to minimize animal pain or discomfort.

### Maintenance of the *S*. *mansoni* life cycle

A Puerto Rican strain of *S*. *mansoni* was maintained by passage through albino *Biomphalaria glabrata*, as the intermediate host, and ICR (CD-1) outbred female mice as definitive host as previously described [25]. Mice infected 7–8 weeks previously with single sex or double sex cercariae were euthanized with intraperitoneal injections of Tiletamine/Zolazepam (800 mg/kg) + Xylazine (100 mg/kg) and adult parasites were harvested by reversed perfusion of the hepatic portal system and mesenteric veins [27]. Juvenile worms were obtained from mice 28 days after infection.

### Preparation of parasites, viability assays and egg counts

Cercariae were converted to newly transformed schistosomula by mechanical transformation using an optimized version of the protocol of Brink *et al*., 1977 [28], previously described by Protasio *et al*. [29] and adapted in our laboratory [25].

The schistosomula viability assay was carried out as previously described [25]. Briefly, compounds dissolved in DMSO were transferred to 384-well, black, tissue culture plates using the acoustic droplet ejection technology (ATS-100, EDC Biosystems, USA). DMSO alone and gambogic acid (10 μM) were used as high and low control in each plate. A suspension of schistosomula in complete DMEM medium (without phenol red) was transferred to assay plates by a multidrop dispenser (Thermo Fisher, USA) in the number of about 100 schistosomula per well in a final volume of 30 μl. After 24 hours incubation at 37°C and 5% CO_2_, a volume of 30 μl of CellTiter-GLO reagent (CTG) (Promega, USA) was added resulting in the generation of a luminescence signal proportional to the amount of ATP present in the well. Sample luminescence levels (proportional to ATP levels) were detected 30 minutes after CTG addition and quantified as RLU (Relative Luminescence Unit) by a charge-coupled device (CCD)-based detector (ViewLux, PerkinElmer USA). Screening and potency results were evaluated by GraphPad (Prism, USA).

Worms recovered from infected mice at 28 days (juvenile worms) or 7–8 weeks (adult worms) post-infection were cultured in DMEM (with phenol red) complete tissue culture medium at 37°C in 5% CO_2_ atmosphere. For all treatments, 5–10 males or couples were incubated with selected compounds at the indicated concentrations and cultured in 3–5 ml of complete tissue culture medium for up to 7 days, unless otherwise stated as previously described [30]. The compound was given to parasites *in vitro* only once without medium addition and/or replacement.

During the culture time, survival was monitored daily under a Leica MZ12 stereomicroscope and viability scored as previously described, based on phenotypical changes like motility and general appearance (tegumental damage and darkness, gut peristalsis and morphology, plate attachment) Briefly, the type and number of phenotypic responses were recorded into a ‘severity score’ ranging from 0 (severely compromised) to 3 (no effect) as previously described [25,30]. The following phenotype scoring criteria were used: 3 = plate-attached, good movements, clear; 2 = slower or diminished movements, darkening, minor tegumental damages; 1 = movements are heavily slowered, dark, tegument is heavily damaged; 0 = dead, lack of any movements. For each sample the following formula was used:

$$\frac{\Sigma \;\left(\text{worm}\;\text{scores}\right)}{number\;of\;worms}$$

Data were expressed as % severity score (viability) relative to DMSO. All tests were repeated at least three times.

Images were recorded using with an Olympus BX41 microscope served by an Olympus SP-350 camera (for adult worms) or an Olympus AX70 fluorescence microscope supported by an Olympus XM10 camera with the Olympus CellSens Standard 1.8.1 software (for eggs, spermatozoa, oocytes and vitelline cells in the worm culture media).

The number of eggs produced by all worm couples was counted at day 3 using an inverted LEICA DM IL microscope.

### Carmine-red staining

Carmine-red staining was performed essentially as previously described [30]. Briefly, adult worms were fixed for at least 24 hours in AFA (95% ethanol at 70%, 3% formaldehyde at 37%, 2% glacial acetic acid) at room temperature, stained for 30 minutes with 2.5% hydrochloric carmine-red and then de-stained by several washes in acidic alcohol (70% EtOH at 100%, 2.5% HCl at 37% and 27.5% double-distilled H_2_O) until no more color was released by the samples. Next, samples were dehydrated for 2 min in 90%, 30 sec in 100% ethanol and worms preserved in Canada balsam on glass slides. Images were taken on an Olympus FV1200 confocal laser scanning microscope using an UPlanFLN 40X immersion oil objective (NA = 1.30) with optical pinhole at 1AU and a multiline argon laser at 488 nm as excitation source. The images were collected as a single stack.

### Statistical analysis

All statistical tests were performed using GraphPad Prism version 6.0c software (San Diego, CA, USA). The data are shown as mean ± standard error of the mean (SEM) or ± standard deviation (SD) as indicated. Differences observed in the *in vitro* assays were analyzed by Student’s t-test. For all experiments, p-values < 0.05 were considered to be statistically significant.

## Results

### Hit identification and confirmation

**Fig 1:**
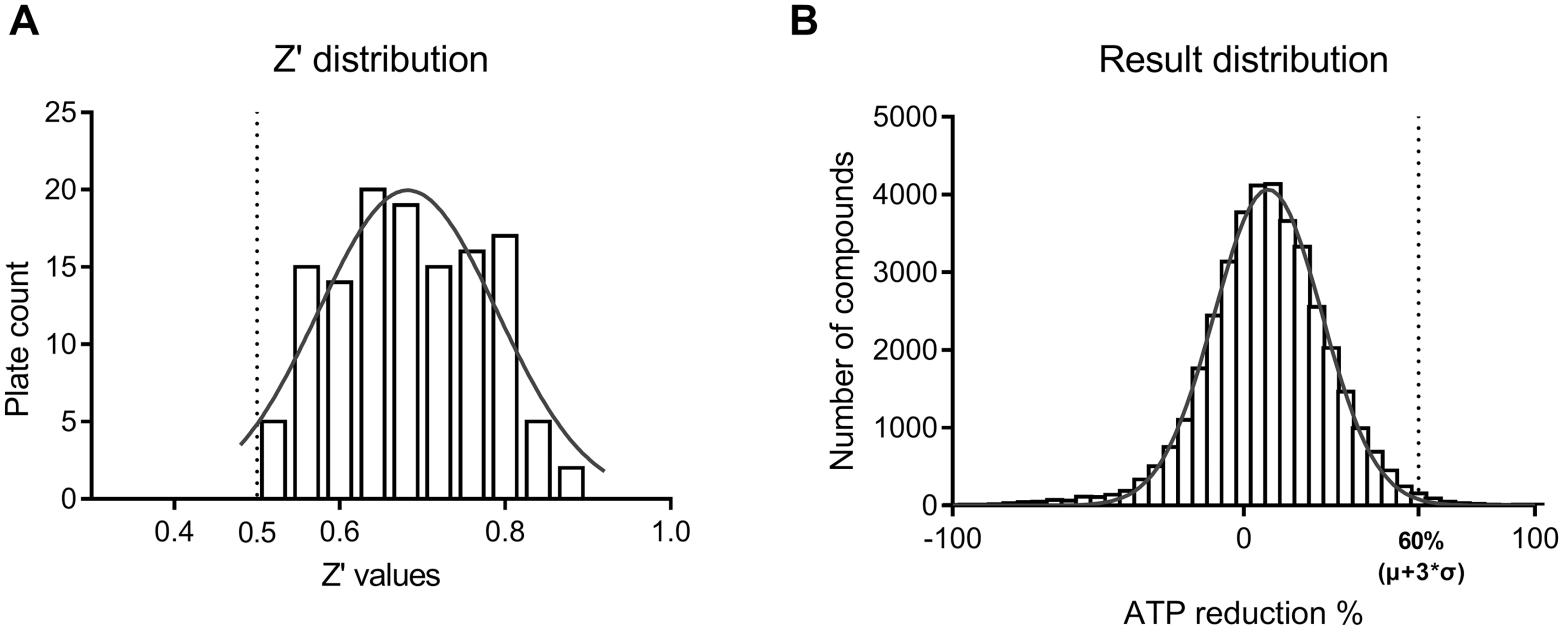
Schistosomula HTS screening. (A) Distribution of Z’ values across all tested plates (128) and Gaussian fitting of binned Z’ values. The dotted vertical line is the 0.5 threshold which is commonly assumed as the lowest value indicating a robust assay (B) Distribution of the tested compound (38, 811) activity expressed as ATP reduction % (death of schistosomula) with respect to DMSO (0%) and gambogic acid 10 μM (100%). The solid curve represents the Gaussian fitting of the binned values. The dotted vertical line is the average plus three times the standard deviation of all compounds (60%), which was set as positivity threshold.

A compound collection comprising 38,811 molecules was screened at a single concentration of 10 μM using the schistosomula viability assay previously described [25]. The screening was carried out in multiple batches, according to the production of schistosomula, with the average number of plates per run being 10. In order to determine the quality of the results, the Z’ value [31] was calculated for each tested plate using the vehicle (DMSO) dispensed wells as negative control and gambogic acid dispensed wells as positive control. All plate Z’ was found to be above the 0.5 threshold which is commonly considered the lowest acceptable value for a robust assay (Fig 1A). The activity of each tested compound was calculated as percentage of ATP reduction against the vehicle (0%) and gambogic acid (100%), thus representing the percentage of dead schistosomula. This normalization allowed compensation for the schistosomula batch-to-batch variations. The compound activity distribution was found to be Gaussian, as a consequence the positivity threshold was set to 60%, which is the average sample activity plus three times the standard deviation (Fig 1B).

**Fig 2:**
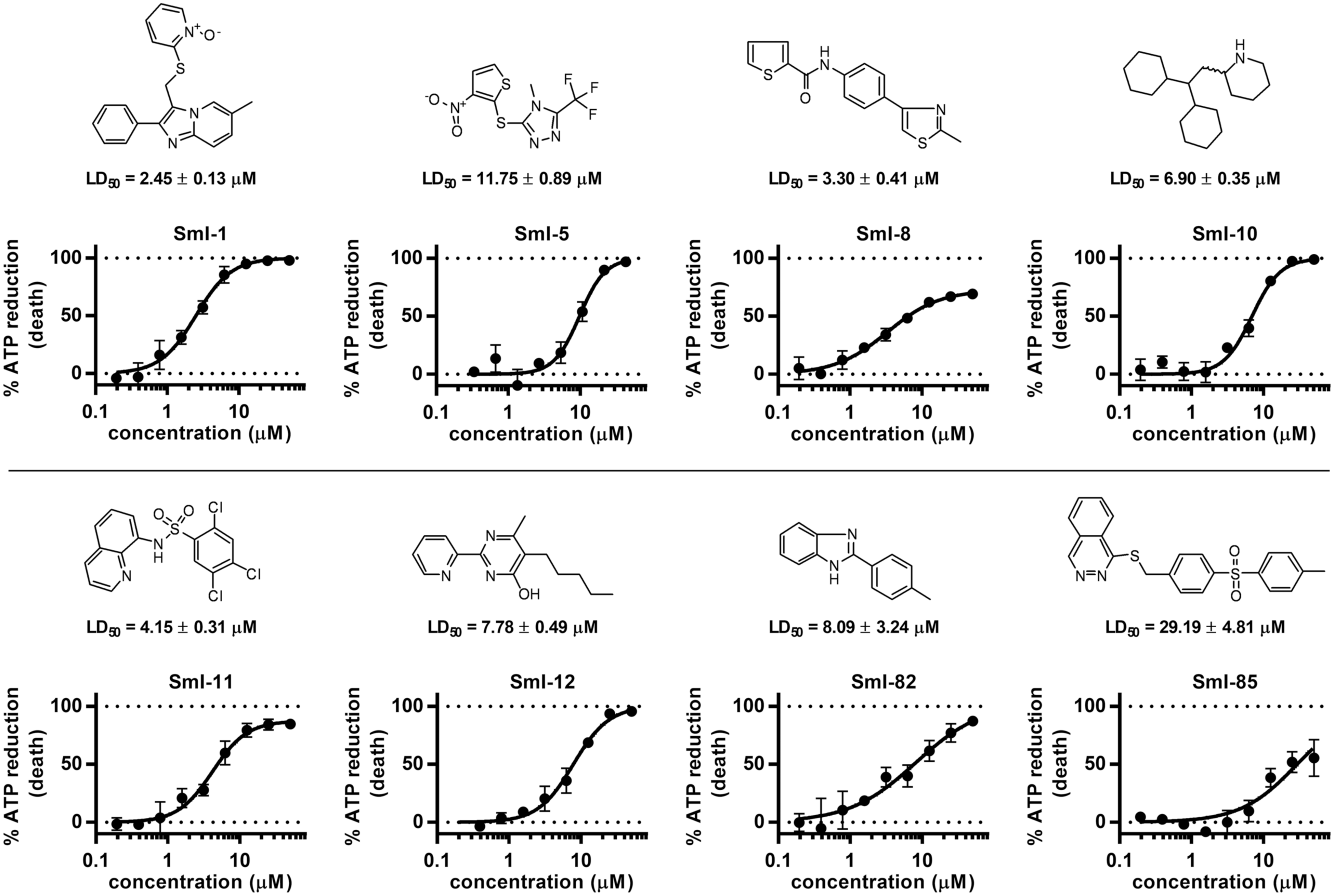
Activity of selected hit compounds on schistosomula. For each hit compound dose response curves of schistosomula viability assay are reported. The % ATP reduction (death of schistosomula) plotted on the y-axis is normalized against DMSO (0%) and gambogic acid 10 μM (100%). Each point represents the average and standard deviation of three independent experiments.

By using this threshold, 275 active compounds were identified (0.7% hit rate). It has to be noted that the average compound activity is greater than 0 and the activity distribution is rather broad. These aspects will be commented on in the discussion section. The 275 hits from the HTS were subjected to quality control by LC-MS in order to check compound identity and purity (acceptable purity criteria set to be > 90% peak area in the diode array trace). Sixteen compounds were discarded at this stage as they failed to pass the QC. Hit selection was performed using a clustering approach based on the Taylor Butina algorithm [32], a non-hierarchical clustering method that ensures that each cluster contains molecules with a certain cut-off (or threshold) distance from a central compound. Circular fingerprints with radius 2 and 2048 bits were generated using the RDKit software [33], with the purpose of generating a similarity matrix based on a Tanimoto index [26,33]. The effective number of neighbours for each molecule was calculated based on the Tanimoto level (0.8) used for clustering. This procedure gave a collection of 80 clusters of which 30 were singletons. Eighty centroids were selected together with 30 compounds picked randomly from the most populated clusters. The final set of 110 compounds was tested again in an independent schistosomula dose response curve assay (40 nM-50 μM) and from this screen 22 compounds were confirmed to be active with LD_50_ ranging from 5 to 40 μM. After visual inspection eight compounds were selected to be further profiled. Fresh powders of these eight compounds were repurchased/resynthesized and retested on schistosomula (Fig 2). The previously found LD_50_’s were confirmed for all eight compounds.

### Identification of multi-stages schistosomicidal compounds

**Fig 3:**
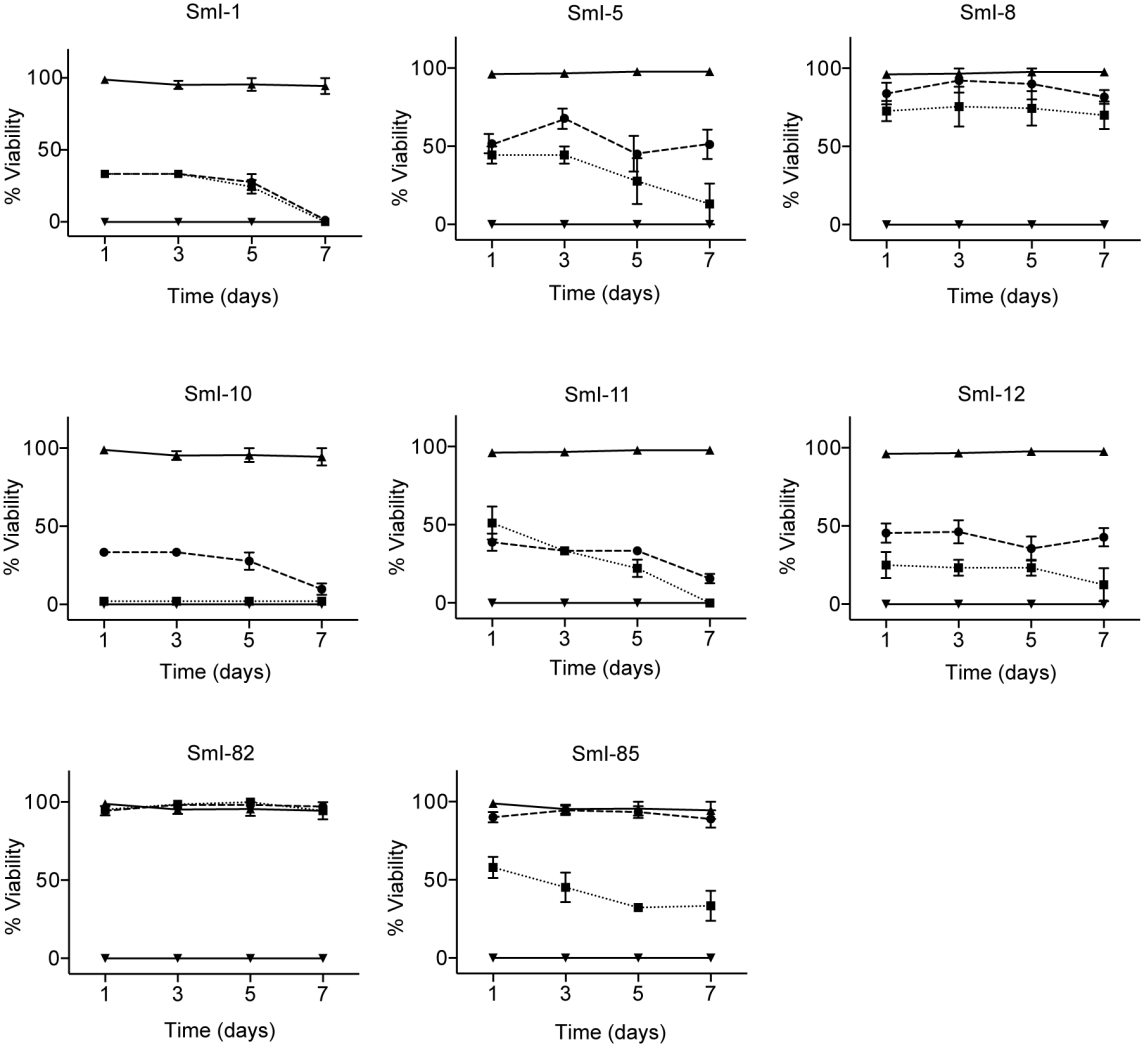
Activity of selected hit compounds on adult *S*. *mansoni* worms. Adult male worms (7–8 weeks old) were incubated with the indicated compounds at the concentration of 10 μM (solid circle) and 20 μM (solid square) and viability of parasites scored at different time points (x-axis) as described under methods. DMSO (vehicle, solid triangle) and gambogic acid (solid inverted triangle) were used respectively as negative and positive control. Each point represents the average ± SEM of three independent experiments.

In order to investigate the efficacy of the eight selected compounds on other *S*. *mansoni* developmental stages, survival assays and bright-field microscopy analyses were performed on adult male worms. All compounds but SmI-8 and SmI-82 negatively influenced adult worms viability; SmI-1, SmI-10 (perhexiline maleate), and SmI-11 showed the highest effect being the most active compounds at 10 μM (Fig 3). Indeed SmI-1 and SmI-11 were able to induce complete parasites death 7 days after a single compound treatment, an effect similar to that previously reported for perhexiline maleate (SmI-10) [30] (Fig 3).

**Fig 4:**
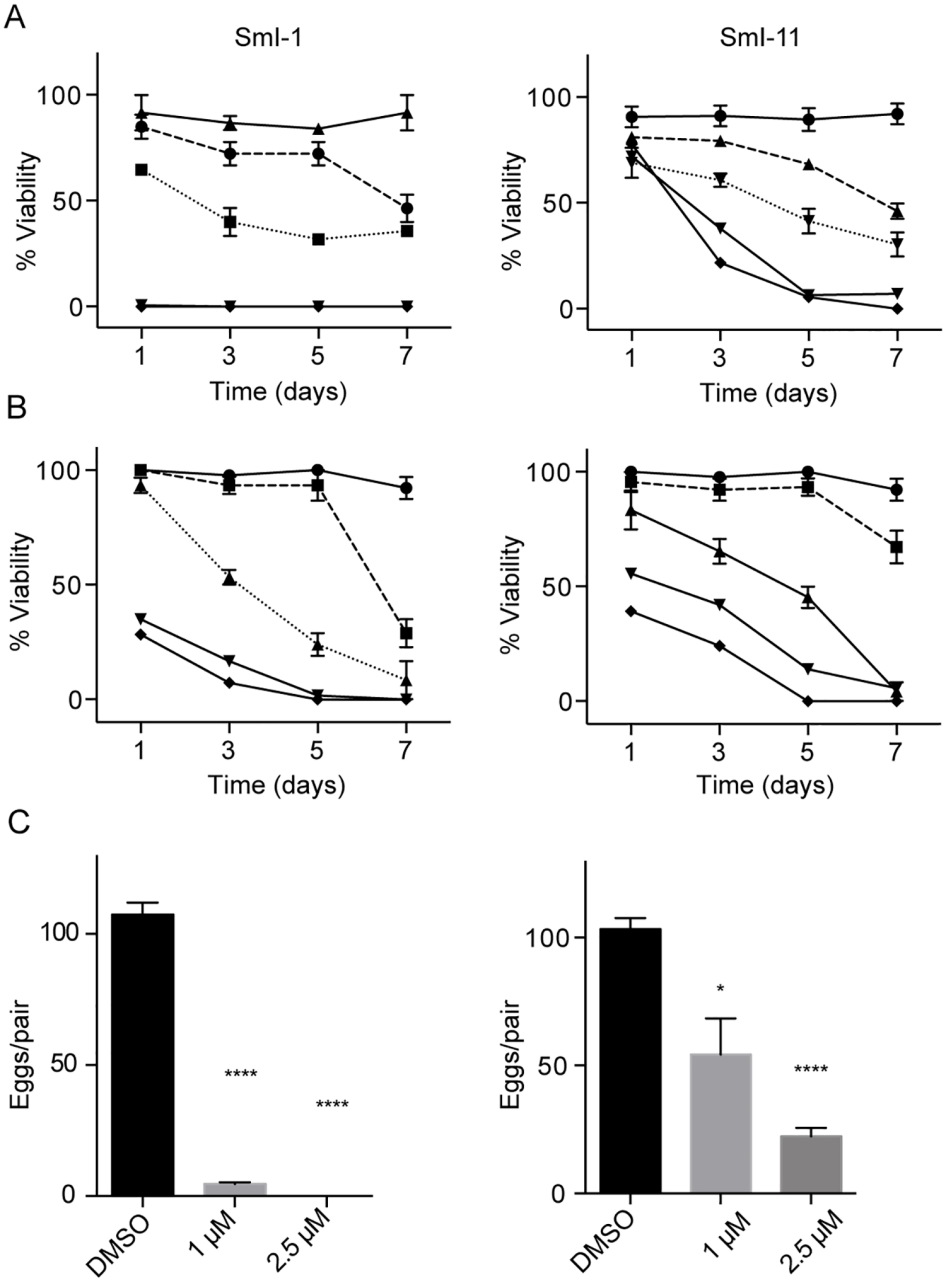
Effects of SmI-1 and SmI-11 compounds on *S*. *mansoni* juvenile worms and adult pairs viability and egg production *in vitro*. Viability curves of juvenile worms (4 weeks old) (A) and adult worms pairs (7–8 weeks old) (B) incubated with the SmI-1 and SmI-11 compounds. Worm viability was assessed as described in the methods section. Parasites were cultivated in presence of increasing concentrations of both compounds as follow: 2.5 μM (solid square), 5 μM (solid triangle), 10 μM (solid inverted triangle), and 20 μM (solid diamond). DMSO was used as negative control (solid circle). C) Total egg counts laid by parasites treated during 72 hours with different sub-lethal doses of SmI-1 and SmI-11 normalized to worm couples. The mean data ± SEM of three independent experiments are shown. The levels of statistical significance are indicated above bars; *p-value < 0.05, ****p-value < 0.0001, Student's *t* test.

Importantly, SmI-1 and SmI-11 were highly effective also on juvenile *S*. *mansoni* worms (4 weeks old) (Fig 4A) and mature paired parasites (7–8 weeks old) (Fig 4B). In particular at the concentration of 10 μM, SmI-1 and SmI-11 were able to induce parasites death in juvenile worms respectively 24 hours and 5 days post treatment (Fig 4A) and in mature couples at 5 and 7 days after treatment using 5 μM concentration (Fig 4B).

Such impact on all parasites stages viability has been previously reported also for the compound perhexiline maleate (SmI-10) [30].

Importantly at sub-lethal concentrations (1 and 2.5 μM), even though not significantly affecting viability or parasites pairing, both compounds caused marked reduction in the number of eggs laid by worm pairs 3 days upon treatment (Fig 4C).

### A pharmacophore approach to identify multi-stage schistosomicidal compounds

**Fig 5:**
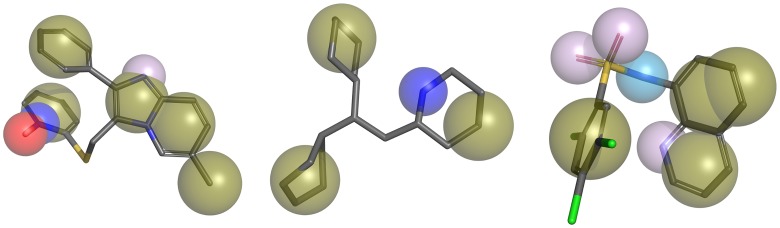
Pharmacophore models used in virtual screening. Code colours: aromatic and lipophilic (HYBL) features are represented by solid dark green spheres, hydrogen bond donor HDON features are represented by light blue spheres, hydrogen bond acceptor HACC features are represented by purple spheres, positive charge centers POSC represented in blue, and negative charge centers NEGC represented in red. 3D pharmacophore models based on SmI-11, SmI-10, and SmI-1. Hydrogen atoms are not shown for clarity.

Ligand-based molecular modelling approaches are generally used when a reliable target structure is not available from which to derive the structural requirements important for activity. Pharmacophore modeling belongs to this kind of methodology and has been widely used in drug discovery [34]. To identify novel scaffolds by using the three identified hits, SmI-11, SmI-10 (perhexiline maleate), and SmI-1, 3D pharmacophore models were developed. A 3D structure for each compound was generated using energy minimization and the resultant minimized structures were used as starting points for subsequent conformational analysis using a multi-objective genetic algorithm implemented in BALLOON software [35,36]. Unique low energy conformations within 10 kcal/mole of the corresponding global energy minimum were collected for each molecule. Pharmacophore models were then generated using Align-it software [37]. On the basis of the structural characteristics of compounds SmI-11, SmI-10, and SmI-1 aromatic and lipophilic features, hydrogen bond donor, hydrogen bond acceptor and positive charge were considered for model development. The generated pharmacophore models were visually evaluated through the generation of molecular maps as described in Fig 5.

**Fig 6:**
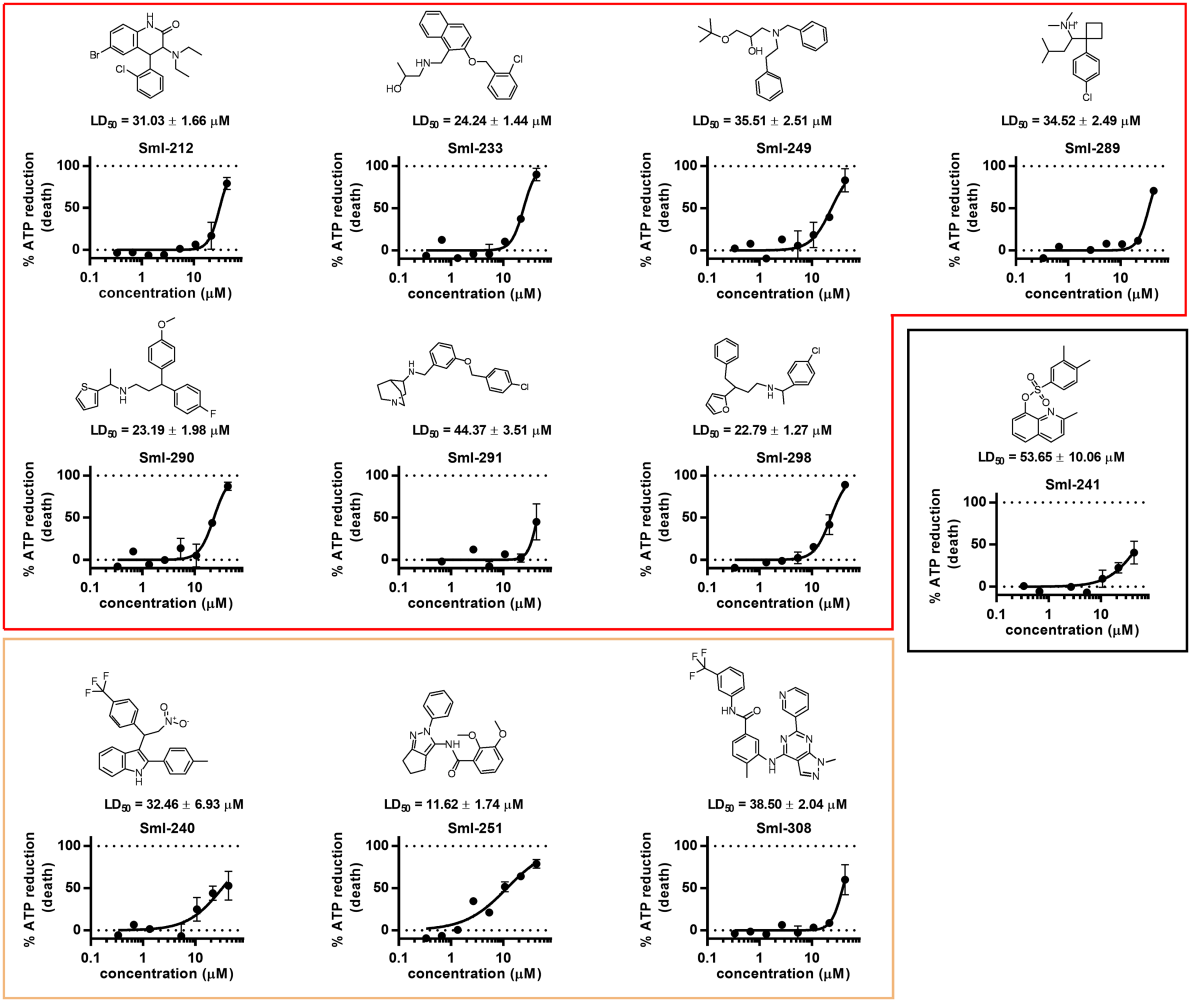
Activity on schistosomula of compounds selected by a pharmacophore approach. Compounds were grouped based on the parent compound. Compounds within the red frame were identified based on the SmI-10 pharmacophore, compounds within the orange frame were identified based on the SmI-1 pahrmacophore, and the compound in the black frame was identified by the SmI-11 pharmacophore. For each compound dose response curves on schistosomula viability assay are reported. The % ATP reduction (schistosomula death) plotted on the y-axis is normalized between DMSO (0%) and gambogic acid 10 μM (100%). Each point represents the average and standard deviations of three independent experiments.

A database search to find compounds matching the pharmacophores was conducted using an expanded version of IRBM’s internal library consisting of 40,000 molecules. Results were ranked on the basis of the Tversky similarity index [38] and the number of features in the corresponding phamacophoric model. Top 130 candidates were selected and assayed on schistosomula: 11 resulted with a good LD_50_ and were further profiled. The resulted compounds consisted of seven hits derived from the SmI-10 (perhexiline maleate) model, one from the SmI-11 model (sulphonamide), and three based on the pharmacophoric model of SmI-1 (Imidazo(1,2-a)pyridine) (Fig 6).

**Fig 7:**
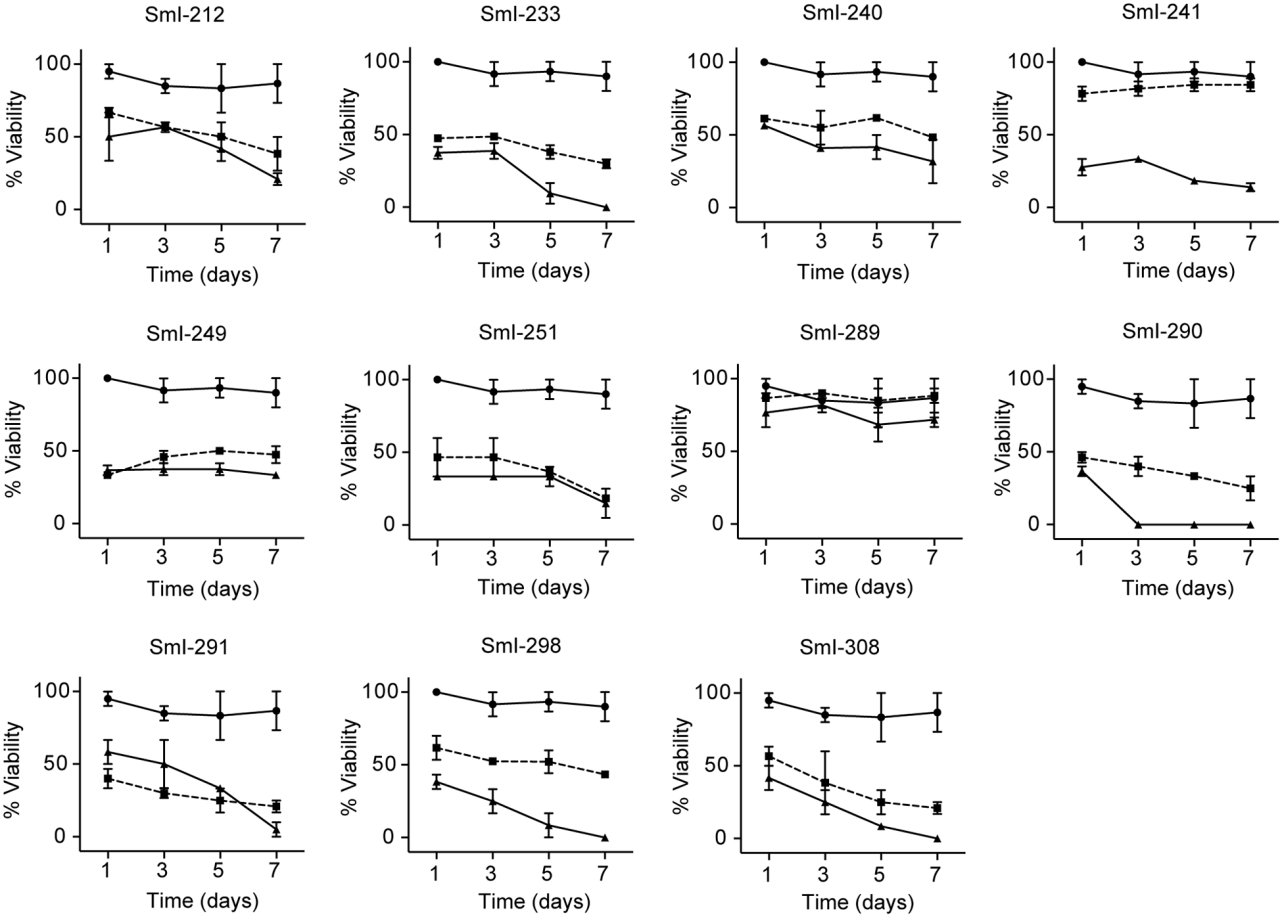
Activity on adult *S*. *mansoni* worms of compounds selected by a pharmacophore approach. Viability curves of adult male worms (7–8 weeks old) incubated with the indicated compounds at the concentration of 10 μM (solid square) and 20 μM (solid triangle). The viability of parasites scored at different time points (x-axis) as described under methods. DMSO (solid circle) was used as control. Each point represents the average ± SEM of three independent experiments.

Next, to test their ability to impact worms survival the new 11 compounds were all assayed on adult male worms at the concentrations of 10 and 20 μM with five of them (SmI-233, SmI-251, SmI-290, SmI-291, and SmI-308) being active. In particular at the concentration of 20 μM we found less than 50% survival rate in worms 72 h upon treatment and almost complete death occurring at day 7 (Fig 7).

**Fig 8:**
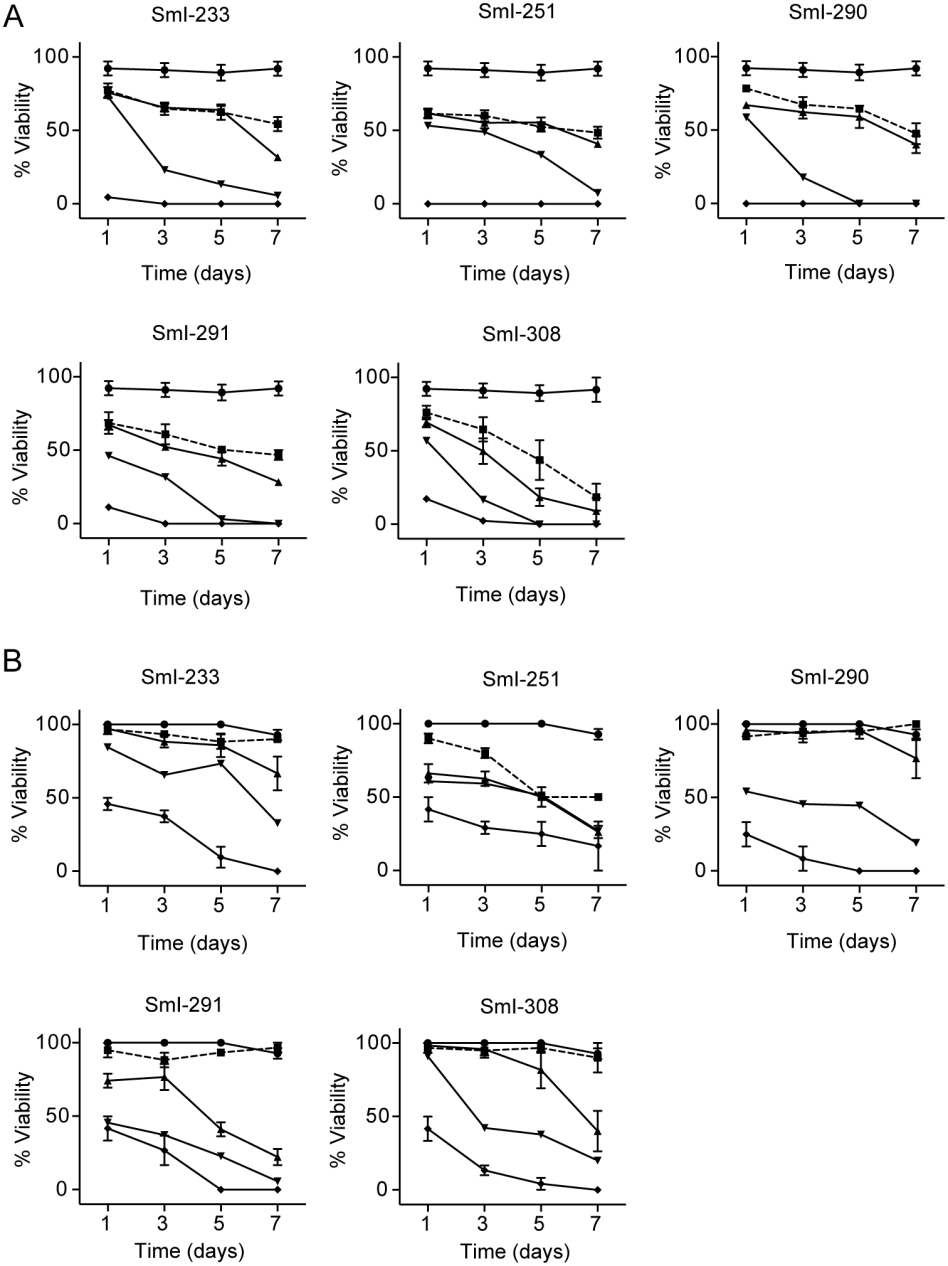
Activity on *S*. *mansoni* juvenile worms and adult pairs of compounds selected by a pharmacophore approach. Viability curves of juvenile worms (A) and adult pairs (B) incubated with the indicated compounds using the following concentrations: 2.5 μM (solid square), 5 μM (solid triangle), 10 μM (inverted solid triangle), and 20 μM (solid diamond). DMSO (solid circle) was used as control. The mean data ± SEM of three independent experiments are shown.

Importantly all the selected compounds were also very active on both juvenile worms (4 weeks old) (Fig 8A) and mature pairs (7–8 weeks old) (Fig 8B). In particular at the concentration of 20 μM all five compounds induced death of all juvenile parasites in 24 h and at the concentration of 10 μM less than 30% survival rate 72 h upon treatment with almost complete death occurring at day 5 (SmI-233, SmI-290, SmI-291, and SmI-308) or day 7 (SmI-251). The SmI-308 compound was also very effective on juvenile worms even at lower concentrations (2.5–5 μM). With respect to adult worm pairs, at the concentration of 20 μM all five compounds induced almost complete death at day 5 (SmI-233, SmI-290, SmI-291 and SmI-308) or day 7 (SmI-251). The compounds also demonstrated to be very active on pairs treated at the concentration of 10 μM at day 7.

**Fig 9:**
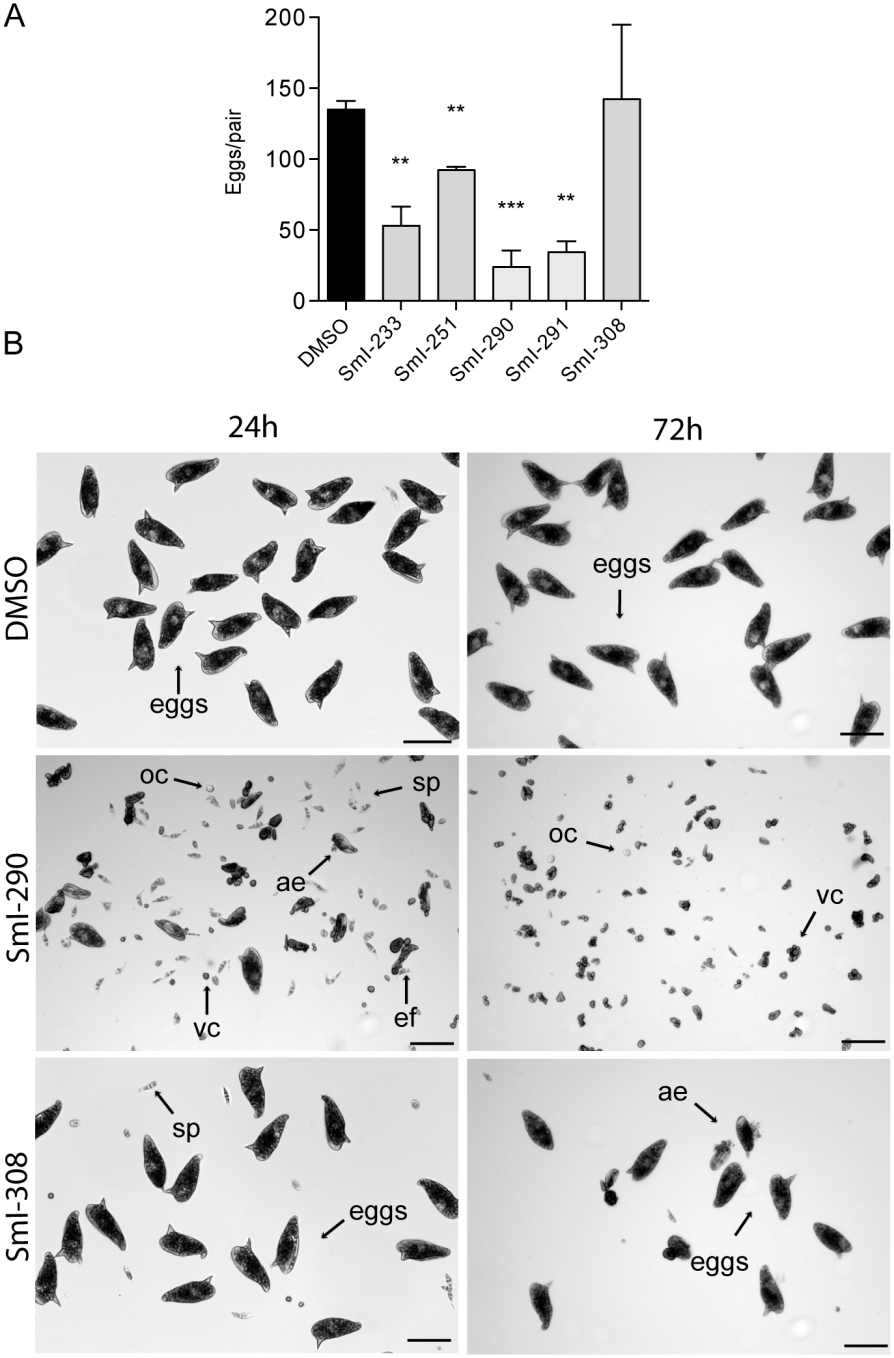
Activity on the production of eggs of compounds selected by a pharmacophore approach. A) Histograms showing the total egg counts normalized to worm couples treated for 72 hours with sub-lethal doses of the indicated compounds. The levels of statistical significance are indicated above bars: ** p-values ≤ 0.01, *** p-values ≤0.001, Student's *t* test. B) Representative images of eggs, oocytes (oc), spermatozoa (sp), vitelline cells (vc), eggshell fragments (ef), and abnormal eggs (ae) in the tissue culture medium of worm couples treated with DMSO or 5 μM of SmI-290, or SmI-308 at 24 and 72 hours. Tissue culture medium and compounds were freshly replaced every 24 hours. Scale bars: 100 μm.

Moreover using sub-lethal doses (5 or 2.5 μM) all the compounds, with the only exception of compound SmI-308, had a strong impact on egg production during 72 hours of treatment (Fig 9A). Intriguingly, the compounds causing egg production impairment also induced the release of a number of oocytes, spermatozoa, vitelline cells, eggshell fragments, and abnormal eggs in the tissue culture medium while no effect was recorded for the compound SmI-308. An example of the tissue culture media observed 24 and 72 hours after treatment with compounds SmI-290 and SmI-308 is shown in Fig 9B.

Compared to the other compounds a quite peculiar phenotype was observed in parasites treated with the SmI-308 compound. Despite the fact that parasites did not seem to be affected in terms of viability, pairing status, egg production, and germ cells release during the first 72 hours at sub-lethal doses, bright-field microscopy showed a general swelling of both male and female worms with a marked gut dilatation (S1 Fig).

### Morphological alterations of compounds-treated parasites

**Fig 10:**
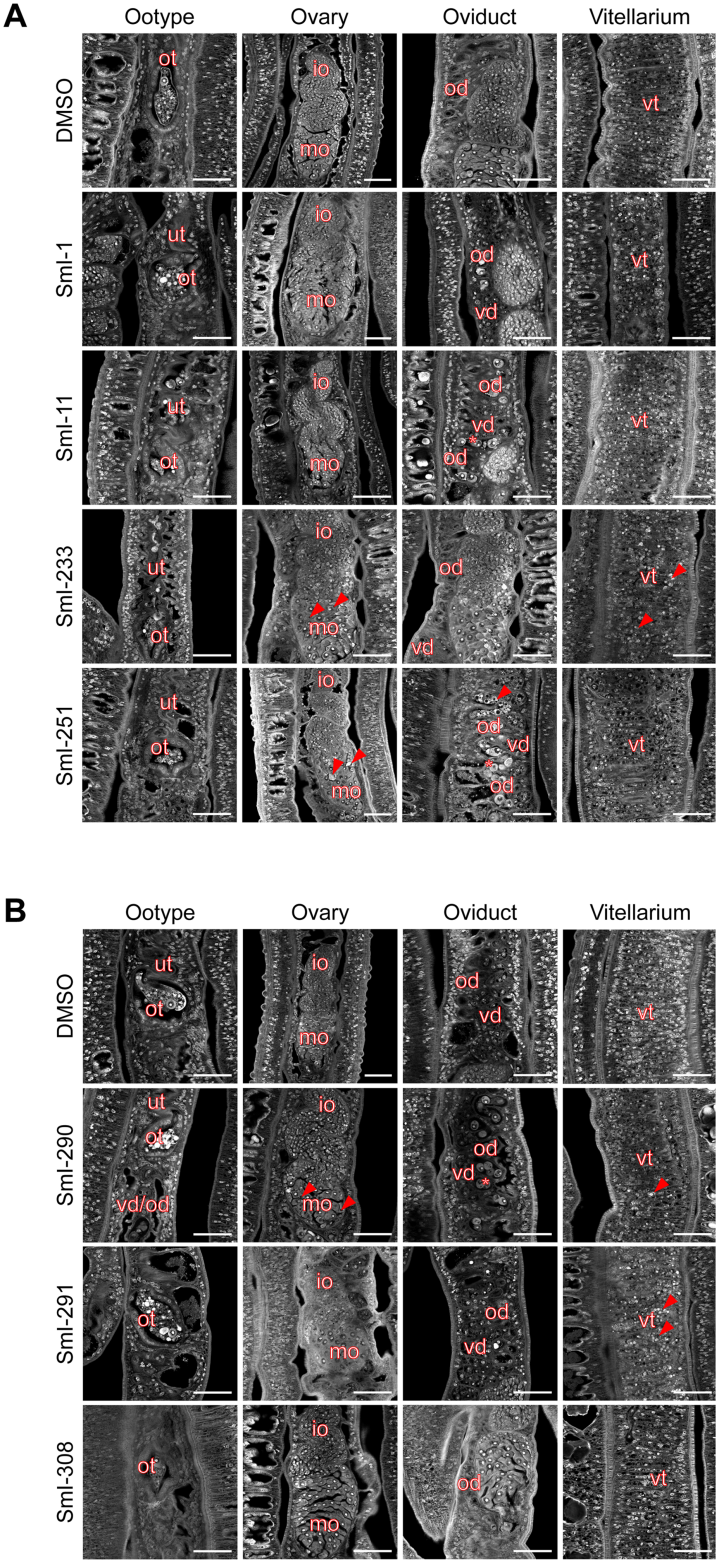
Effect of selected schistosomicidal compounds on the phenotype of *S*. *mansoni* worm pairs: Female reproductive organs alterations. Representative confocal laser microscopy images of *S*. *mansoni* female reproductive system (ootype, ovary, oviduct and vitellarium). Worm pairs were treated for 72 hours with the indicated compounds: A) SmI-1 and SmI-11 at 2.5 μM concentration; SmI-233 and SmI-251 at 5 μM concentration; B) SmI-290 and SmI-308 at 5 μM concentration and SmI-291 at 2.5 μM concentration (ootype, oviduct and vitellarium images) and 5 μM concentration (ovary). Worm pairs were stained with carmine-red as described in the methods section. In Figure 10A and 10B immature oocytes (io), mature oocytes (mo), uterus (ut), ootype (ot), vitelline duct (vd), oviduct (od), vitello-oviduct (vd/od). Arrows indicate degenerated cells and asterisk mature oocytes engulfment within the oviduct. Scale bars: 50 μm.

In order to characterize the phenotypic alterations induced by compound treatment in more detail, carmine-red staining and confocal laser scanning microscopy analysis were performed. Remarkably, with the unique exception of compound SmI-308, all selected compound- treated worms showed impairment in egg formation (Fig 10A and 10B). Indeed, in the ootype of female parasites treated for 72 hours with SmI-1, SmI-11, SmI-233, SmI-251, SmI-290 (5 μM), and SmI-291 (2.5 μM) we did not detect any mature eggs. Moreover, the ootype and often also the uterus showed the presence of disorganized oocytes, vitelline cells and intense carmine-red positive elements thought likely to be eggshell components. A similar ootype phenotype was observed in worms treated with perhexiline maleate (5 μM) 72 hours after treatment (S2c Fig). On the contrary the ootype of worms treated with the SmI-308 compound or DMSO (vehicle) contained healthy eggs ready to be laid (Fig 10A and 10B). The overall structure of the ovaries seemed not to be affected thus retaining the normal organization with small immature oocytes (IO) in the anterior part and large mature oocytes (MO) in the posterior part. Also the vitellarium seemed to preserve its structure. However the ovaries of females treated with compounds SmI-233, SmI-251, SmI-290, and SmI-291 (5 μM) contained oocytes with alterations. In particular the ovary of worms treated with compounds SmI-233, SmI-290 and, to a lesser extent, with SmI-291 showed black spots in MO likely due to degeneration. In SmI-251-treated females aggregates of degenerate cells in the anterior part containing MO and a marked degeneration of IO were observed in the ovary. A strong degeneration of IO was also observed in parasites treated with SmI-233 and SmI-291 compounds treatment (Fig 10A and 10B). Moreover oviducts of parasites treated with compounds SmI-11, SmI-251, and SmI-290 showed a remarkable engulfment and defects of trafficking of MO towards the ootype. This phenotype was also sporadically observed in some worms treated with SmI-1, SmI-233, and SmI-291 compounds (Fig 10A and 10B) and in perhexiline maleate-treated samples (S2 Fig). On the contrary we did not detect oocytes in the oviduct of female parasites treated with compound SmI-308 or DMSO (vehicle) (Fig 10A and 10B). Finally female worms of pairs treated for 72 hours with SmI-233, SmI-290 (5 μM) and SmI-291 (2.5 μM) also showed an increased number of degenerated cells in vitelline follicles (Fig 10A and 10B).

**Fig 11:**
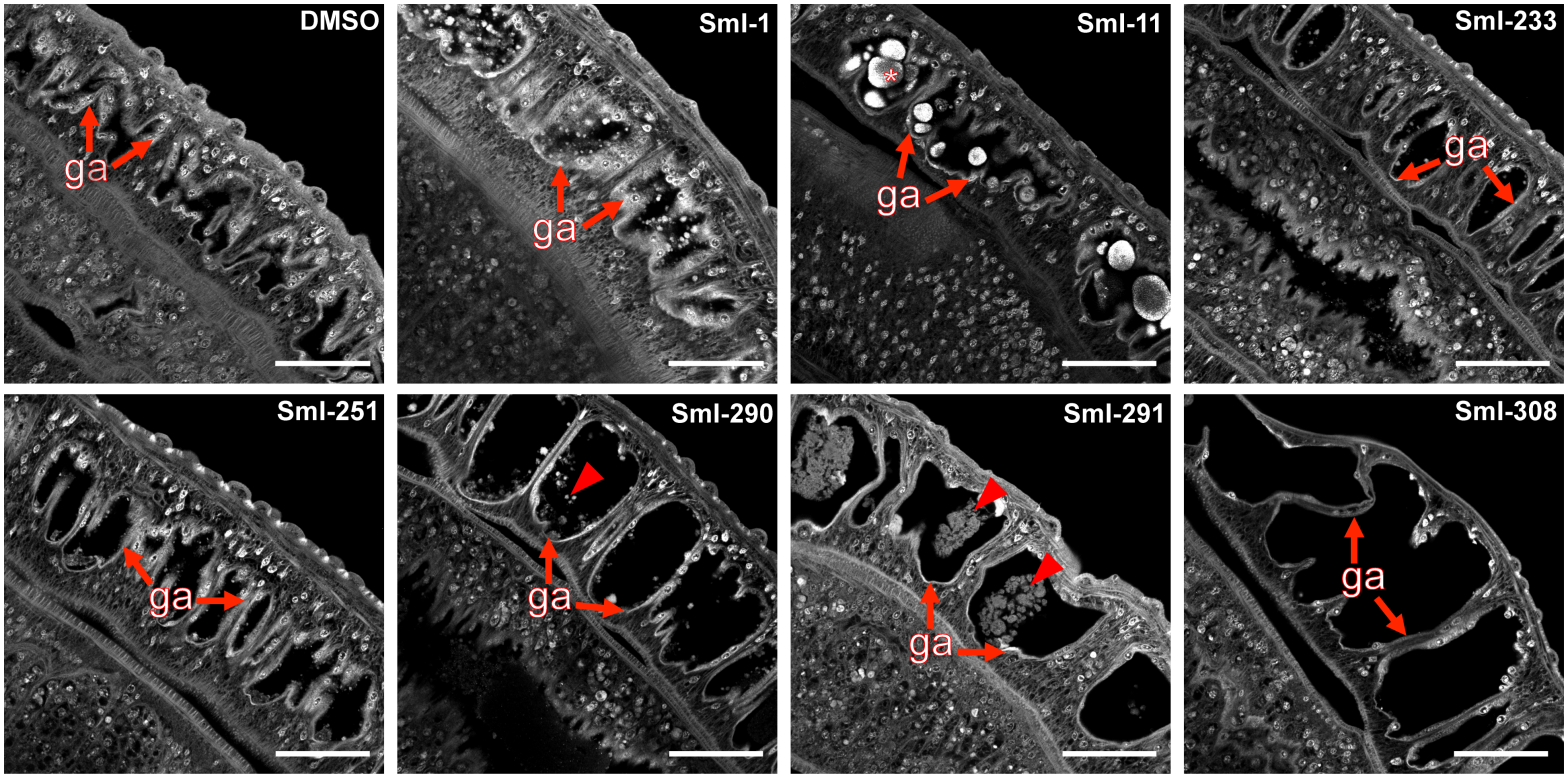
Effect of selected schistosomicidal compounds on *S*. *mansoni* worm pairs: gut alterations. Representative confocal laser microscopy images of the gut of *S*. *mansoni* male worms. Worm pairs were treated for 72 hours with the indicated compounds: SmI-1, SmI-11, SmI-291 at 2.5 μM concentration; SmI-233, SmI-251, SmI-290 and SmI-308 at 5 μM concentration and stained with carmine-red. In the gut images, the gastrodermis (ga), carmine-red aggregates (asterisk), and gastrodermis detachment (arrows) are indicated. Scale bars: 50 μm.

In addition, although phenotype consistency was not always seen for all worms within a test sample, we observed that all compounds with the only exception of SmI-1 led to gut dilation with detachment of the gastrodermis and accumulation of carmine-red positive particle aggregates in the lumen (Fig 11). This alteration was especially notable for compounds SmI-290, SmI-291 and SmI-308 (Fig 11). The morphology and cellularity of the testicular lobes were similar in males of pairs treated with all selected compounds or DMSO with the exception of those treated with SmI-308 that showed cavities in the testicular lobes (S3 Fig), cavities also present in the ovary of females. Mature sperm was present in all seminal vesicles and receptacles of male and female treated parasites.

## Discussion and conclusion

In modern drug discovery, the process of lead identification involves both the screening of a compound collection against a target or a complete organism and the validation of a set of hits having acceptable activity to fight the disease. To this aim small organisms such as schistosomula can be employed using *in vitro* assays to screen large set of compounds in an automated, objective and high-throughput manner [25]. The initial screening using schistosomula can offer both advantages and disadvantages: the use of such a small and handy stage represent an useful tool for increased throughput and improved automation but the screen workflow with schistosomula need to be validated in the other stages of the parasite.

Further phenotypic screen in the juvenile and adult parasite developmental stages as well as egg formation and production assessment are required in order to identify novel multi-stage schistosomicidal compounds with dual effects on both parasites viability and egg production impairment.

In an attempt to search for new chemical compounds for the treatment of schistosomiasis having these features, combination of high throughput library screening on schistosomula, a pharmacophore approach and phenotypic analyses on both juvenile, known to be less sensitive to PZQ compared to mature egg-laying adults [10–13], and adult parasites was successfully used for the identification of eight novel multi-stage schistosomicidal compounds, including perhexiline maleate that we previously described [30].

As reported in the results section, the HTS campaign was carried out in multiple batches, with compound throughput depending on the yield of each lot of the schistosomula production. On average ten 384-well plates were carried out per run. As a consequence we found extremely important to ensure consistent parasite production and minimize potential sources of contaminations. Once all HTS data were analysed they were found to be Gaussian distributed confirming that there is no bias in the compound collection or in the assay itself. The distribution of the results was rather broad likely due to random errors arising from both the influence of multiple assay runs and the limited number of parasites per well. In addition, the average of all HTS values was greater than zero suggesting that DMSO controls, which are located in a corner of the plate, may have slightly suffered from evaporation.

In follow up to the HTS screen our approach to profile 300 hit compounds was supported by computational chemistry. A combination of clustering and retesting identified 22 *bona fide* inhibitors from which eight hits were selected for further work. In macroscopic terms these eight compounds can be grouped into 3 categories: i) biphenyl analogs (SmI-1, SmI-8, SmI-12, SmI-82); ii) biaryl analogs/compounds containing two linked aryl rings (SmI-5, SmI-11, SmI-85); iii) non-aryl analogs (SmI-10). One compound from each of these categories (SmI-1, SmI-11 and SmI-10) was selected taking into account its effect on viability reduction of both juvenile and adult parasites as wells as egg production impairment. Computational analysis was used to generate more detailed pharmacophore models to identify the key elements of the structures responsible for biological activity. Use of these pharmacophores to identify further potential schistosomicidal compounds proved successful. From the 130 compounds that based on the pharmacophores were selected for assay on schistosomula, 11 confirmed hits were obtained (Fig 6). These included five analogs that were of interest and that retained activity against juvenile and adult worms.

Importantly, all selected compounds, with the only exception of SmI-308, were also able to interfere with the egg production process when used at sub-lethal doses. Along with survival data, the egg impairment caused by the selected compounds represents an outstanding aspect in fighting the spreading of schistosomisiasis as the egg production represents a key component for the transmission and immunopathology of the disease.

In addition, morphological studies by confocal microscopy analyses highlighted strong characteristic egg-associated phenotypes for seven out of eight hits. A lack of eggshell formation and an absence of mature eggs in the ootype associated to degeneration of immature and/or mature oocytes and vitelline cells and a remarkable engulfment of MO and vitelline cells, appeared to be some common features among these seven compounds (Fig 10). Defects in the egg machinery with production of abnormal eggs were observed for all compound-treatments with the only exception of compound SmI-308. On the other hand, parasites treated with SmI-308 showed a completely different phenotype characterized by a remarkable gut enlargement with tegumental invaginations and oedema-like swellings of the body (S1 Fig) also detectable in the reproductive organs (ovary and testis) (S3 Fig) similar to the one recently described by others with arylmethylamino steroids compounds [39]. Defects in the ootype, with formation of dysplastic eggs have been shown before as consequences of knocking-down the activity of protein kinases [40–43] as well as treatment with derivatives of biarylalkyl carboxylic acid [44]. Alteration of the gastrodermis, that we describe here, has been detected with several other schistosomicidal compounds including mefloquine [45,46] or artemether [47] or derivatives of biarylalkyl carboxylic acid [44] or arylmethylamino steroids compounds [39].

Eggshell assemblage and egg formation occurring in schistosomes [48] is a complex process also epigenetically regulated [49]. Further understanding of their regulatory molecular mechanisms is valuable since disruption of these processes may provide leads for intervention with drugs for controlling schistosomiasis.

We can conclude that the pharmacophore approach can represent an important tool in new hits identification process. It is encouraging that we have been able to identify a number of compounds with IC50 <10 μM against all developmental stages of the parasite definitive host and with potency against juvenile worms higher than PZQ. It is interesting that some morphological alterations associated to the treatment seem to be related to defects in the egg machinery and even thought the potential targets are still unknown, the phenotypes observed with these novel schistosomicidal compounds could drive investigation toward some common mechanisms, such as on genes and signaling pathways involved in eggshell assemblage and eggs formation.

## Supporting information

S1 Fig Effects of SmI-308 compound on male worm phenotype. Bright-field microscopy showing a peculiar phenotype of adult male worms treated for 7 days with SmI-308 at 10 μM. Scale bars in the upper and lower panels are 500 μm and 25 μm respectively.
(TIF)

S2 Fig Effects of perhexiline maleate on egg formation and oocytes trafficking. Representative confocal laser microscopy images of worm pairs treated with perhexiline maleate (2.5 μM) for 72 hours and stained with carmine-red. a) Uterus containing vitelline cells and carmine-red positive elements; b) Disorganized vitelline cells and oocyte in the ootype; c) Oviduct engulfed with oocytes and cellular debris. Uterus (ut), ootype (ot), ovary (ov), vitelline duct (vd), oviduct (od). Scale bars: 50 μm.
(TIF)

S3 Fig Effects of the SmI-308 compound on testis and ovary morphology. Representative confocal laser microscopy images of testis and ovary of worm pairs treated for 72 hours with the SmI-308 compound at 5 μM concentration and stained with carmine-red. Seminal vescicle (sv), immature oocytes (io), mature oocytes (mo), cavities (asteriscs). Scale bars: 50 μm.
(TIF)

## References

[1] Global Health Estimates. http://www.who.int/healthinfo.

[2] Schistosomiasis. http://www.who.int/mediacentre/factsheets/fs115/en/.

[3] Basch PF. Schistosomes: Development, Reproduction, and Host Relations. Oxford University Press; New York: 1991.

[4] Popiel I, Basch PF. Reproductive development of female *Schistosoma mansoni* (Digenea: Schistosomatidae) following bisexual pairing of worms and worm segments. J Exp Zool. 1984; 232: 141–150. doi: 10.1002/jez.1402320117 6502090 10.1002/jez.1402320117

[5] Loverde PT, Chen L. Schistosome female reproductive development. Parasitol Today. 1991; 7: 303–308. 15463396 10.1016/0169-4758(91)90263-n

[6] Kunz W. Schistosome male-female interaction: induction of germ-cell differentiation. Trends Parasitol. 2001; 17: 227–231. 11323306 10.1016/s1471-4922(01)01893-1

[7] Pearce EJ, MacDonald AS. The immunobiology of schistosomiasis. Nat Rev Immunol. 2002; 2: 499–511. doi: 10.1038/nri843 12094224 10.1038/nri843

[8] Cioli D, Pica-Mattoccia L, Basso A, Guidi A. Schistosomiasis control: praziquantel forever? Mol Biochem Parasitol. 2014; 195: 23–29. doi: 10.1016/j.molbiopara.2014.06.002 24955523 10.1016/j.molbiopara.2014.06.002

[9] Doenhoff MJ, Cioli D, Utzinger J. Praziquantel: mechanisms of action, resistance and new derivatives for schistosomiasis. Curr Opin Infect Dis. 2008; 21: 659–667. doi: 10.1097/QCO.0b013e328318978f 18978535 10.1097/QCO.0b013e328318978f

[10] Pica-Mattoccia L, Cioli D. Sex- and stage-related sensitivity of *Schistosoma mansoni* to *in vivo* and *in vitro* praziquantel treatment. Int J Parasitol. 2004; 34: 527–533. doi: 10.1016/j.ijpara.2003.12.003 15013742 10.1016/j.ijpara.2003.12.003

[11] Xiao SH, Catto BA, Webster LT Jr. Effects of praziquantel on different developmental stages of *Schistosoma mansoni in vitro* and *in vivo*. J Infect Dis. 1985; 151: 1130–1137. 3998507 10.1093/infdis/151.6.1130

[12] Sabah AA, Fletcher C, Webbe G, Doenhoff MJ. *Schistosoma mansoni*: chemotherapy of infections of different ages. Exp Parasitol. 1986; 61: 294–303. 3086114 10.1016/0014-4894(86)90184-0

[13] Aragon AD, Imani RA, Blackburn VR, Cupit PM, Melman SD, et al Towards an understanding of the mechanism of action of praziquantel. Mol Biochem Parasitol.2009; 164: 57–65. doi: 10.1016/j.molbiopara.2008.11.007 19100294 10.1016/j.molbiopara.2008.11.007 PMC2886009

[14] Dabo A, Doucoure B, Koita O, Diallo M, Kouriba B, et al [Reinfection with *Schistosoma haematobium* and *mansoni* despite repeated praziquantel office treatment in Niger, Mali]. Med Trop (Mars).2000; 60: 351–355. 11436587

[15] N'Goran EK, Utzinger J, N'Guessan AN, Muller I, Zamble K, et al Reinfection with *Schistosoma haematobium* following school-based chemotherapy with praziquantel in four highly endemic villages in Cote d'Ivoire. Trop Med Int Health.2001; 6: 817–825. 11679130 10.1046/j.1365-3156.2001.00785.x

[16] Melman SD, Steinauer ML, Cunningham C, Kubatko LS, Mwangi IN, et al Reduced susceptibility to praziquantel among naturally occurring Kenyan isolates of *Schistosoma mansoni*. PLoS Negl Trop Dis. 2009; 3: e504 doi: 10.1371/journal.pntd.0000504 19688043 10.1371/journal.pntd.0000504 PMC2721635

[17] Gryseels B, Mbaye A, De Vlas SJ, Stelma FF, Guisse F, et al Are poor responses to praziquantel for the treatment of *Schistosoma mansoni* infections in Senegal due to resistance? An overview of the evidence. Trop Med Int Health. 2001; 6: 864–873. 11703840 10.1046/j.1365-3156.2001.00811.x

[18] Cioli D, Botros SS, Wheatcroft-Francklow K, Mbaye A, Southgate V, et al Determination of ED50 values for praziquantel in praziquantel-resistant and -susceptible *Schistosoma mansoni* isolates. Int J Parasitol. 2004; 34: 979–987. doi: 10.1016/j.ijpara.2004.05.001 15217737 10.1016/j.ijpara.2004.05.001

[19] Mwangi IN, Sanchez MC, Mkoji GM, Agola LE, Runo SM, et al Praziquantel sensitivity of Kenyan *Schistosoma mansoni* isolates and the generation of a laboratory strain with reduced susceptibility to the drug. Int J Parasitol Drugs Drug Resist. 2014; 4: 296–300. doi: 10.1016/j.ijpddr.2014.09.006 25516840 10.1016/j.ijpddr.2014.09.006 PMC4266778

[20] Fallon PG, Doenhoff MJ. Drug-resistant schistosomiasis: resistance to praziquantel and oxamniquine induced in *Schistosoma mansoni* in mice is drug specific. Am J Trop Med Hyg. 1994; 51: 83–88. 8059919 10.4269/ajtmh.1994.51.83

[21] Ismail MM, Taha SA, Farghaly AM, el-Azony AS. Laboratory induced resistance to praziquantel in experimental schistosomiasis. J Egypt Soc Parasitol. 1994; 24: 685–695. 7844435

[22] Sabra AN, Botros SS. Response of *Schistosoma mansoni* isolates having different drug sensitivity to praziquantel over several life cycle passages with and without therapeutic pressure. J Parasitol. 2008; 94: 537–541. doi: 10.1645/GE-1297.1 18564758 10.1645/GE-1297.1

[23] Liang YS, Li HJ, Dai JR, Wang W, Qu GL, et al [Studies on resistance of *Schistosoma* to praziquantel XIII resistance of *Schistosoma japonicum* to praziquantel is experimentally induced in laboratory]. Zhongguo Xue Xi Chong Bing Fang Zhi Za Zhi. 2011; 23: 605–610. 22379812

[24] Couto FF, Coelho PM, Araujo N, Kusel JR, Katz N, et al *Schistosoma mansoni*: a method for inducing resistance to praziquantel using infected *Biomphalaria glabrata* snails. Mem Inst Oswaldo Cruz. 2011; 106: 153–157. 21537673 10.1590/s0074-02762011000200006

[25] Lalli C, Guidi A, Gennari N, Altamura S, Bresciani A, et al Development and validation of a luminescence-based, medium-throughput assay for drug screening in *Schistosoma mansoni*. PLoS Negl Trop Dis. 2015; 9: e0003484 doi: 10.1371/journal.pntd.0003484 25635836 10.1371/journal.pntd.0003484 PMC4312041

[26] Rogers DJ, Tanimoto TT. A Computer Program for Classifying Plants. Science. 1960; 132: 1115–1118. doi: 10.1126/science.132.3434.1115 17790723 10.1126/science.132.3434.1115

[27] Pellegrino J, Siqueira AF. [A perfusion technic for recovery of *Schistosoma mansoni* from experimentally infected guinea pigs]. Rev Bras Malariol Doencas Trop. 1956; 8: 589–597. 13494879

[28] Brink LH, McLaren DJ, Smithers SR. *Schistosoma mansoni*: a comparative study of artificially transformed schistosomula and schistosomula recovered after cercarial penetration of isolated skin. Parasitology.1977;74: 73–86. 320543 10.1017/s0031182000047545

[29] Protasio AV, Dunne DW, Berriman M. Comparative study of transcriptome profiles of mechanical- and skin-transformed *Schistosoma mansoni* schistosomula. PLoS Negl Trop Dis. 2013; 7: e2091 doi: 10.1371/journal.pntd.0002091 23516644 10.1371/journal.pntd.0002091 PMC3597483

[30] Guidi A, Lalli C, Perlas E, Bolasco G, Nibbio M, et al Discovery and Characterization of Novel Anti-schistosomal Properties of the Anti-anginal Drug, Perhexiline and Its Impact on *Schistosoma mansoni* Male and Female Reproductive Systems. PLoS Negl Trop Dis. 2016; 10: e0004928 doi: 10.1371/journal.pntd.0004928 27518281 10.1371/journal.pntd.0004928 PMC4982595

[31] Zhang JH, Chung TD, Oldenburg KR. A Simple Statistical Parameter for Use in Evaluation and Validation of High Throughput Screening Assays. J Biomol Screen. 1999; 4: 67–73. doi: 10.1177/108705719900400206 10838414 10.1177/108705719900400206

[32] Butina D. Unsupervised Data Base Clustering Based on Daylight's Fingerprint and Tanimoto Similarity: A Fast and Automated Way To Cluster Small and Large Data Sets. J Chem Inf Comput Sci. 1999; 39(4): 747–750

[33] Landrum G. RDKit: Open-source cheminformatics. 2006; http://www.rdkit.org 2006.

[34] Caporuscio F, Tafi A. Pharmacophore modelling: a forty year old approach and its modern synergies. Curr Med Chem. 2011; 18: 2543–2553. 21568893 10.2174/092986711795933669

[35] Vainio MJ, Johnson MS. Generating conformer ensembles using a multiobjective genetic algorithm. J Chem Inf Model. 2007; 47: 2462–2474. doi: 10.1021/ci6005646 17892278 10.1021/ci6005646

[36] Puranen JS, Vainio MJ, Johnson MS. Accurate conformation-dependent molecular electrostatic potentials for high-throughput in silico drug discovery. J Comput Chem. 2010; 31: 1722–1732. doi: 10.1002/jcc.21460 20020481 10.1002/jcc.21460

[37] Taminau J, Thijs G, De Winter H. Pharao: pharmacophore alignment and optimization. J Mol Graph Model. 2008; 27: 161–169. doi: 10.1016/j.jmgm.2008.04.003 18485770 10.1016/j.jmgm.2008.04.003

[38] Tversky A. Features of similarity. Psychological review. 1977; 84(4): 327–352

[39] Krieg R, Jortzik E, Goetz AA, Blandin S, Wittlin S, et al Arylmethylamino steroids as antiparasitic agents. Nat Commun. 2017; 8: 14478 doi: 10.1038/ncomms14478 28211535 10.1038/ncomms14478 PMC5321741

[40] Beckmann S, Buro C, Dissous C, Hirzmann J, Grevelding CG. The Syk kinase SmTK4 of *Schistosoma mansoni* is involved in the regulation of spermatogenesis and oogenesis. PLoS Pathog. 2010; 6: e1000769 doi: 10.1371/journal.ppat.1000769 20169182 10.1371/journal.ppat.1000769 PMC2820527

[41] Beckmann S, Grevelding CG. Imatinib has a fatal impact on morphology, pairing stability and survival of adult *Schistosoma mansoni in vitro*. Int J Parasitol. 2010; 40: 521–526. doi: 10.1016/j.ijpara.2010.01.007 20149792 10.1016/j.ijpara.2010.01.007

[42] Dissous C. Venus Kinase Receptors at the Crossroads of Insulin Signaling: Their Role in Reproduction for Helminths and Insects. Front Endocrinol (Lausanne). 2015; 6: 118. 26284029 10.3389/fendo.2015.00118 PMC4522560

[43] Leutner S, Beckmann S, Grevelding CG. Characterization of the cGMP-dependent protein kinase SmcGK1 of *Schistosoma mansoni*. An Acad Bras Cienc. 2011; 83: 637–648. 21670884 10.1590/s0001-37652011000200023

[44] Blohm AS, Mader P, Quack T, Lu Z, Hahnel S, et al Derivatives of biarylalkyl carboxylic acid induce pleiotropic phenotypes in adult *Schistosoma mansoni in vitro*. Parasitol Res. 2016; 115: 3831–3842. doi: 10.1007/s00436-016-5146-7 27230017 10.1007/s00436-016-5146-7

[45] Manneck T, Haggenmuller Y, Keiser J. Morphological effects and tegumental alterations induced by mefloquine on schistosomula and adult flukes of *Schistosoma mansoni*. Parasitology. 2010; 137: 85–98. doi: 10.1017/S0031182009990965 19814844 10.1017/S0031182009990965

[46] Xiao SH. Mefloquine, a new type of compound against schistosomes and other helminthes in experimental studies. Parasitol Res. 2013; 112: 3723–3740. doi: 10.1007/s00436-013-3559-0 23979493 10.1007/s00436-013-3559-0

[47] Xiao S, Shen B, Utzinger J, Chollet J, Tanner M. Ultrastructural alterations in adult *Schistosoma mansoni* caused by artemether. Mem Inst Oswaldo Cruz. 2002; 97: 717–724. 12219141 10.1590/s0074-02762002000500023

[48] deWalick S, Tielens AG, van Hellemond JJ. *Schistosoma mansoni*: the egg, biosynthesis of the shell and interaction with the host. Exp Parasitol.2012; 132: 7–13. doi: 10.1016/j.exppara.2011.07.018 21840309 10.1016/j.exppara.2011.07.018

[49] Carneiro VC, de Abreu da Silva IC, Torres EJ, Caby S, Lancelot J, et al Epigenetic changes modulate schistosome egg formation and are a novel target for reducing transmission of schistosomiasis. PLoS Pathog. 2014; 10: e1004116 doi: 10.1371/journal.ppat.1004116 24809504 10.1371/journal.ppat.1004116 PMC4014452

