# Supplementary figures and images for: Discovery by organism based high-throughput screening of new multi-stage compounds affecting Schistosoma mansoni viability, egg formation and production
Source: PLoS Negl Trop Dis. 2017 Oct 6;11(10):e0005994. doi: 10.1371/journal.pntd.0005994 (PMC5646872; doi:10.1371/journal.pntd.0005994)

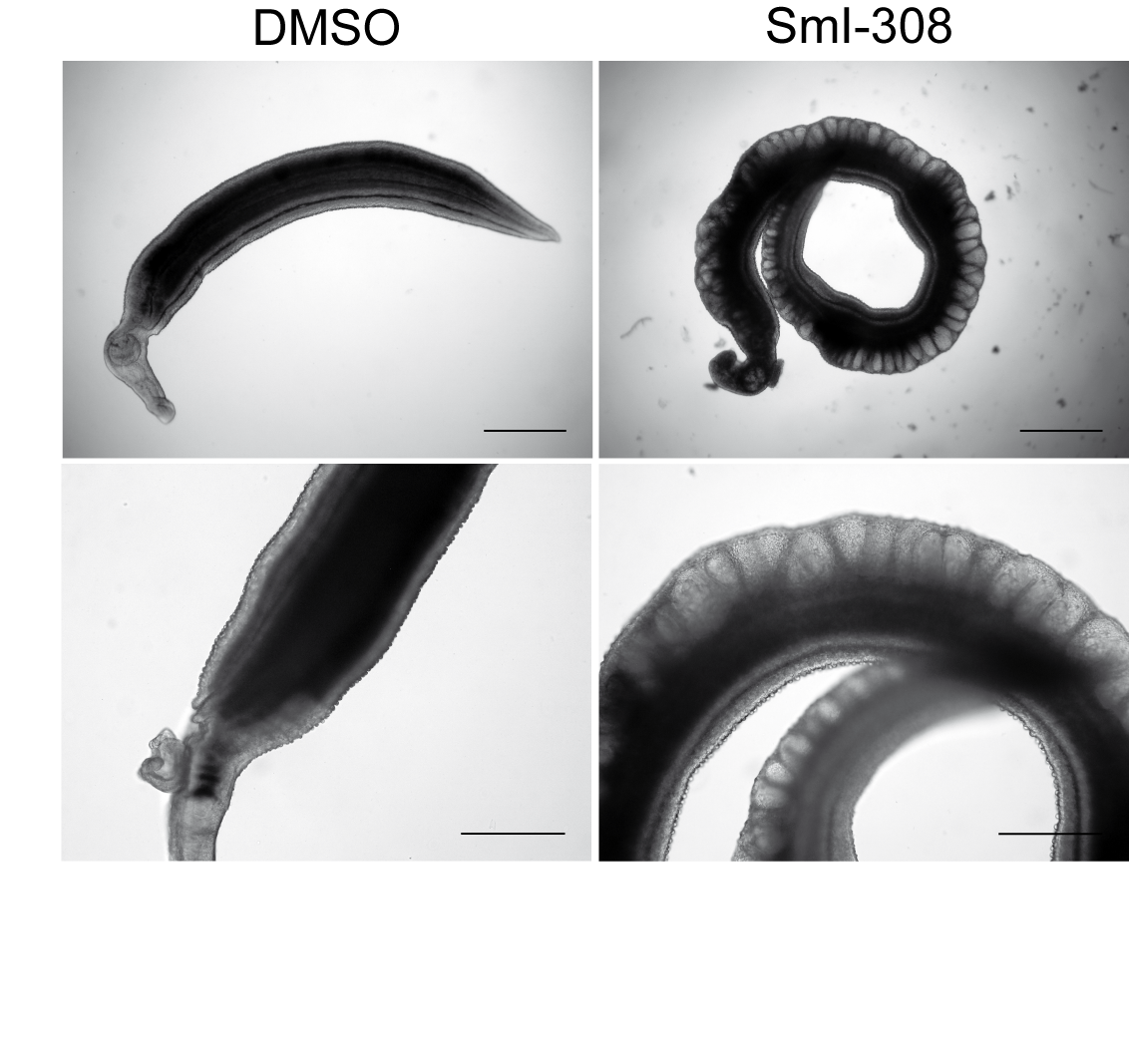

Supplement: S1 Fig — Bright-field microscopy showing a peculiar phenotype of adult male worms treated for 7 days with SmI-308 at 10 μM. Scale bars in the upper and lower panels are 500 μm and 25 μm respectively. (TIF) [file pntd.0005994.s001.tif]

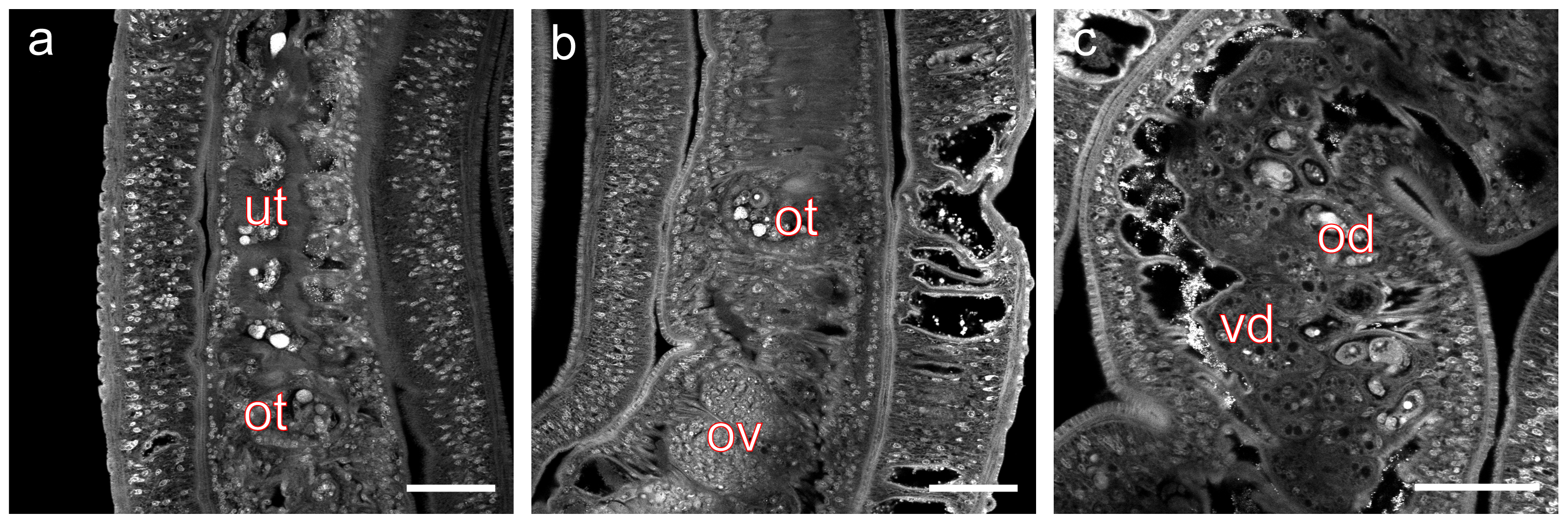

Supplement: S2 Fig — Representative confocal laser microscopy images of worm pairs treated with perhexiline maleate (2.5 μM) for 72 hours and stained with carmine-red. a) Uterus containing vitelline cells and carmine-red positive elements; b) Disorganized vitelline cells and oocyte in the ootype; c) Oviduct engulfed with oocytes and cellular debris. Uterus (ut), ootype (ot), ovary (ov), vitelline duct (vd), oviduct (od). Scale bars: 50 μm. (TIF) [file pntd.0005994.s002.tif]

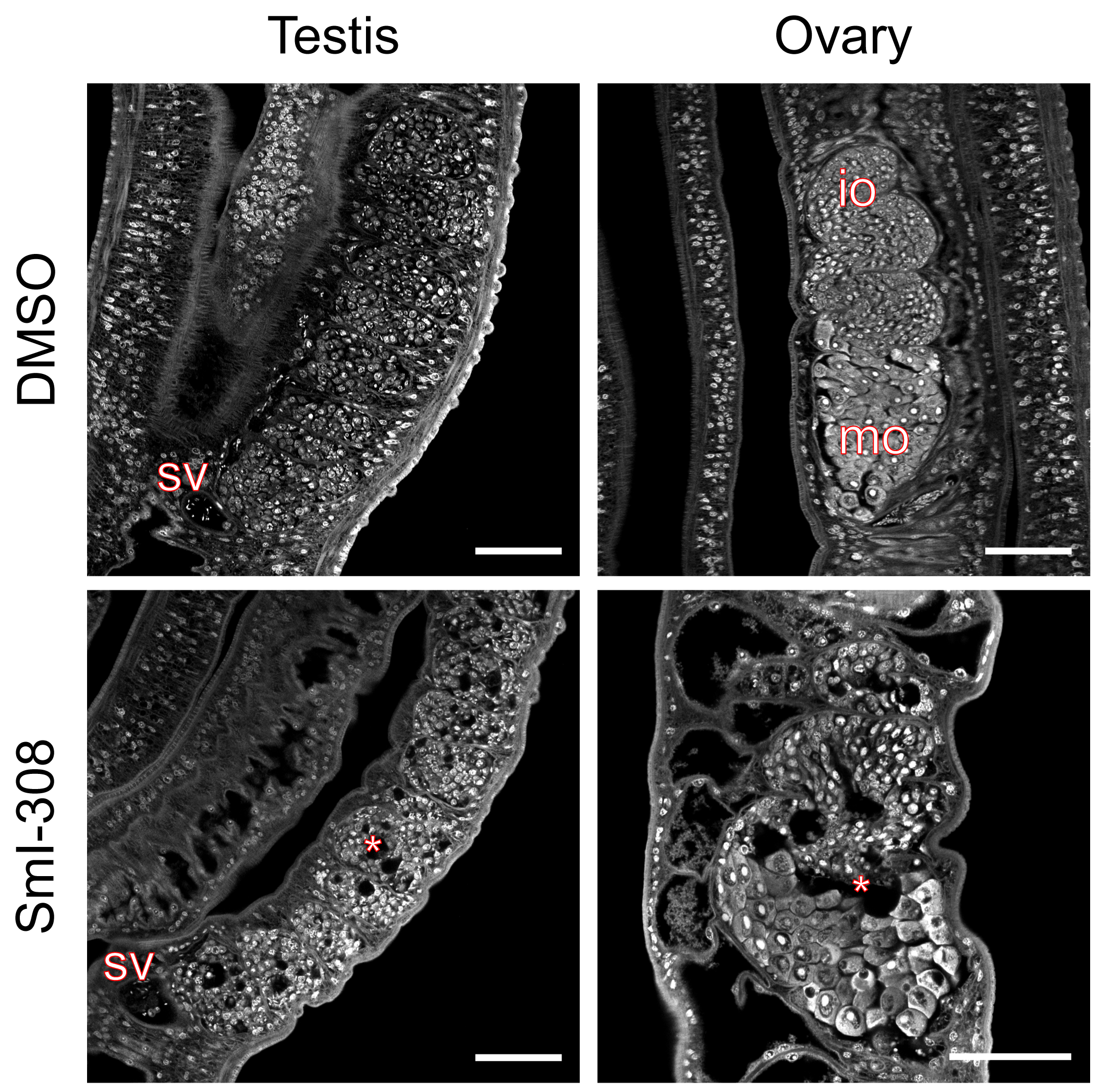

Supplement: S3 Fig — Representative confocal laser microscopy images of testis and ovary of worm pairs treated for 72 hours with the SmI-308 compound at 5 μM concentration and stained with carmine-red. Seminal vescicle (sv), immature oocytes (io), mature oocytes (mo), cavities (asteriscs). Scale bars: 50 μm. (TIF) [file pntd.0005994.s003.tif]
